# Micro-gyms as a catalyst for healthy aging in university and healthcare settings: applications for the Semmelweis-EUniWell Workplace Health Promotion Model Program

**DOI:** 10.1007/s11357-025-01595-9

**Published:** 2025-03-15

**Authors:** Noémi Mózes, Dorottya Árva, David Major, Mónika Fekete, Norbert Dósa, Andrea Lehoczki, Péter Varga, Kata Pártos, Wei Yi Hung, Giorgia Giovannetti, Daniele Vignoli, Beatrix Busse, Mariann Moizs, Iveta Nagyova, Yongjie Yon, György Purebl, Béla Merkely, Róza Ádány, Vince Fazekas-Pongor, Zoltán Ungvári

**Affiliations:** 1 Institute of Preventive Medicine and Public Health, Faculty of Medicine, Semmelweis University, Üllői Út 26, Budapest, 1085 Hungary; 2 Doctoral College - Health Sciences Program, Semmelweis University, Budapest, Hungary; 3 Clinical Center, Semmelweis University, Budapest, Hungary; 4 Department of Economics and Management, University of Florence, Florence, Italy; 5 Department of Statistics, Computer Science, Applications, University of Florence, Florence, Italy; 6 Department of Linguistics, University of Cologne, Cologne, Germany; 7 Ministry of Interior of Hungary, Budapest, Hungary; 8 Department of Social and Behavioural Medicine, Faculty of Medicine, PJ Safarik University in Kosice, Kosice, Slovakia; 9 WHO Regional Office for Europe, Copenhagen, Denmark; 10 Healthy Aging Section, European Public Health Association, Utrecht, The Netherlands; 11 Institute of Behavioral Sciences, Faculty of Medicine, Semmelweis University, Budapest, Hungary; 12 Heart and Vascular Center, Semmelweis University, Budapest, Hungary; 13 HUN-REN-UD Public Health Research Group, Department of Public Health and Epidemiology, Faculty of Medicine, University of Debrecen, Debrecen, Hungary; 14 Vascular Cognitive Impairment and Neurodegeneration Program, Oklahoma Center for Geroscience and Healthy Brain Aging, Department of Neurosurgery, University of Oklahoma Health Sciences Center, Oklahoma City, OK USA; 15 Department of Health Promotion Sciences, College of Public Health, University of Oklahoma Health Sciences Center, Oklahoma City, OK USA; 16 International Training Program in Geroscience, Doctoral College - Health Sciences Program/Institute of Preventive Medicine and Public Health, Semmelweis University, Budapest, Hungary

**Keywords:** Healthy aging, Exercise, Workplace health promotion, Fitness, Well-being, Sedentary lifestyles, Motivational interviewing

## Abstract

Europe is experiencing a significant demographic shift, with aging populations posing economic and social challenges due to increased healthcare costs and a higher prevalence of age-related diseases. Hungary, in particular, faces these challenges acutely due to higher morbidity and mortality rates from a range of chronic age-related diseases and behavioral risk factors. Addressing these issues requires innovative approaches to promote healthy aging. Semmelweis University, the largest healthcare provider and leading health sciences university in the region, is developing a comprehensive healthy aging program. A critical pillar of this program is the Semmelweis-EUniWell Workplace Health Promotion Model Program, a pioneering initiative aimed at tackling unhealthy aging within Hungary’s workforce by leveraging the workplace as a platform for health promotion. Central to this program’s goal of combating sedentary lifestyles—a significant contributor to age-related health issues—is the innovative use of micro-gyms and motivational interviewing. Micro-gyms, with their compact size and accessibility, provide convenient exercise opportunities, while motivational interviewing fosters intrinsic motivation and personalized counseling to encourage sustained physical activity. Through concerted efforts and innovative approaches, including the implementation of micro-gyms, the Semmelweis-EUniWell Workplace Health Promotion Model Program aims to set a benchmark for workplace health promotion, fostering a healthier and more resilient aging population in Hungary. This program not only enhances the well-being of employees at Semmelweis University and its EUniWell partner institutions but also catalyzes broader transformations in workplace health promotion and healthy aging nationwide.

## Introduction

Europe faces a significant demographic shift characterized by aging populations. The proportion of people above the age of 65 was already above 20% both in the European Union (EU) and Hungary in 2023 [1, 2], and this is projected to increase to one-third of the population by 2050 [3]. The progressive aging of the European and Hungarian populations is accompanied by an increase in the overall burden of age-related diseases, such as cardiovascular, cancer, metabolic and neurodegenerative diseases [4–6] and thus poses significant economic and social challenges, like increased healthcare costs and greater demand for the care for the older adults [7]. Hungary is affected by these challenge disproportionally because of its higher occurrence of chronic age-related, non-communicable diseases, and higher prevalence of behavioral risk factors associated with unhealthy aging compared to other countries in Europe [8]. To tackle the situation, innovative approaches are necessary to promote healthy aging and enhance the quality of life for older adults across Europe, and especially in Hungary.

Sedentary lifestyle has become one of the greatest public health concerns globally and is one of the major obstacles of healthy aging. According to an analysis of Eurobarometer data, 36.2% of adults in the EU and 35.5% in Hungary were physically inactive in 2017 [9]. Physical inactivity is associated with several unfavorable conditions, like cardiovascular diseases [10], obesity [11, 12], diabetes [12], and musculoskeletal disorders [13] that directly affect quality of life as these conditions accumulate with ageing. Prolonged inactivity, however, not only impacts physical health of individuals but also contributes to psychological issues such as depression and anxiety known to affect elder population more severely [14–16]. Furthermore, sedentary behavior accelerates the aging process itself [17] by promoting the many hallmarks of aging [18], like telomere attrition [19, 20], epigenetic alterations [21], genomic instability [18], mitochondrial dysfunction [22], or cellular senescence [23]. Recognizing the critical link between physical activity and healthy aging, health promotion interventions are of great importance to reduce sedentary time and increase physical activity. By encouraging individuals to adopt active lifestyles, we can mitigate the hallmarks of aging and reduce the individual’s risk of developing age-related diseases [24, 25].

The workplace is a vital setting where individuals spend a significant portion of their lives, making it an ideal environment for health promotion initiatives. By implementing health-promoting initiatives in the workplace, it is possible to encourage the early adoption of healthier lifestyles and preventive measures, thereby positively influencing the aging process. Recognizing this pivotal role, the Semmelweis-EUniWell Workplace Health Promotion Model Program has been developed to address the most relevant risk factors associated with unhealthy aging. This program is tailored specifically to the employees of Semmelweis University, a leading institution in health sciences and Hungary’s most significant healthcare providers, and includes various interventions aimed at promoting healthy aging. Positioned as a cornerstone within the broader National Healthy Aging Program, the Semmelweis-EUniWell Workplace Health Promotion Model Program focuses on workplace-based interventions to advance healthy aging. By fostering healthier behaviors and environments within the workplace, this program aims to pave the way for a nationwide shift towards a healthier and more resilient aging population in Hungary.

Semmelweis University plays a leadership role in the health arena within the European University for Well-Being (EUniWell), a consortium of European universities dedicated to enhancing the well-being of individuals and society through education, research, and community engagement. The EUniWell consortium includes esteemed institutions such as the University of Birmingham in the UK, Linnaeus University in Sweden, Nantes University in France, the University of Cologne in Germany, the University of Florence in Italy, the University of Konstanz in Germany, Semmelweis University in Hungary, the University of Murcia in Spain, the University of Santiago de Compostela in Spain, and Taras Shevchenko National University of Kyiv in Ukraine.

The Semmelweis-EUniWell Workplace Health Promotion Model Program is specifically designed to share good practices, disseminate knowledge, and foster collaborations among the EUniWell member institutions to innovate and implement effective health promotion strategies. The program emphasizes an evidence-based approach, detailed cost analysis, and support for policy development. By focusing on these key areas, the program aims to foster collaboration between European institutions from different cultural backgrounds, developing complex strategies that incorporate input from these diverse knowledge bases to tackle the ageing-related elements of demographic megatrends impacting the EU. Population aging presents challenges and risks but also offers transformative opportunities to foster inclusive well-being for society as a whole. Through the exchange of knowledge and best practices among the member institutions, the program seeks to create innovative solutions that promote well-being and healthy aging within university settings. Additionally, the program aspires to develop approaches that can be adopted and implemented nationally across each member state, thereby contributing to the broader goal of enhancing public health and well-being throughout Europe.

## Workplace health promotion as a strategy

In developing workplace health promotion programs, it is crucial to adhere to the principles of the healthy setting approach, as outlined in the Ottawa Charter [26]. This approach emphasizes that “health is created and lived by people within the settings of their everyday life; where they learn, work, play, and love.” Given that individuals spend a considerable portion of their weekdays in the workplace, it serves as an ideal setting for promoting the health of the working-age population [27]. Recommendations supporting such initiatives are endorsed by the World Health Organization (WHO), the Centers for Disease Control and Prevention (CDC), and the European Union [27–29]. Additionally, specific guidance has been provided regarding the adaptation of workplace health promotion efforts to accommodate factors such as an aging workforce [28, 29]. The demographic composition of the working-age population is evolving, with a notable increase in the proportion of older adults both in the European Union and Hungary [30]. In 2019, approximately one-fifth of workers were 55 years old or older, with a significant rise observed in the employment of individuals aged 65–69 years, often post-state pension age, compared to 2004 [30].

Workplace health promotion programs hold considerable potential for fostering positive health behavior changes among employees. For example, evidence demonstrates that comprehensive smoke-free policies implemented in the workplace can effectively reduce smoking rates among employees [28]. Similarly, these programs have been also associated with successfully promoting healthier dietary habits, achieving better body mass index outcomes, and increasing levels of physical activity [28, 31, 32]. Strategies such as health education, the establishment of supportive norms for health behaviors, provision of healthy food options, and opportunities for training in diet and physical activity can enhance the effectiveness of these programs [28]. Moreover, workplace health promotion programs may also play a role in preventing mental health disorders among employees [32].

With demographic shifts leading to an aging workforce in Europe, there is a trend towards raising the statutory pension age to address labor market challenges [33]. Despite financial considerations, a substantial portion of individuals already continue to work beyond retirement age, often motivated by non-financial factors such as enjoyment of work [30]. In the landscape of Hungary, the situation is particularly pressing. The country’s population ageing is driven by the reductions in fertility and improvements in survival, typically occurring through the demographic transition. As a result, the workforce reserve that can sustain GDP growth is increasingly found in the 65 + age group. Given these circumstances, it is essential to keep the 65 + population in good health to attract them to the job market. Maintaining a healthy and satisfied older workforce is crucial for the stability and growth of the national economy. Workplace health promotion programs play a pivotal role in this context [28]. By effectively addressing the health needs and preferences of employees, these programs can significantly contribute to maintaining a productive workforce. Health promotion initiatives can help older employees manage chronic conditions, reduce the risk of age-related diseases, and improve their overall quality of life. Furthermore, promoting health within the workplace can foster a sense of well-being and job satisfaction among older employees, encouraging them to remain in the workforce longer. This not only benefits the individuals but also helps mitigate the economic impact of an aging population. By investing in the health of the aging workforce, Hungary can better navigate its demographic challenges and ensure sustained economic growth.

## The Semmelweis-EUniWell workplace health promotion model program

In response to the concerning demographic and epidemiological trends observed in Hungary, a comprehensive and integrated approach is imperative. Semmelweis University, recognizing its pivotal role as the nation’s foremost institution in health sciences, has undertaken an ambitious initiative to introduce a multifaceted Healthy Aging Program. This program spans various research domains, including preclinical, translational, clinical, epidemiological, and public health research [34], with the goal of elucidating the determinants of unhealthy aging. By addressing these determinants through both preventive medicine [35] and public health approaches, the program aims to innovate and develop diverse interventions. These interventions encompass pharmacological, nutraceutical, and lifestyle modifications, all with the objective of optimizing the aging process, mitigating age-related diseases, and ultimately extending the healthy lifespan of Hungary’s aging population.

The Semmelweis-EUniWell Workplace Health Promotion Model Program represents a comprehensive initiative positioned at the confluence of employee well-being and national public health endeavors. By integrating strategies aimed at fostering healthy aging, this program directly aligns with Hungary’s National Healthy Aging Program. The overarching vision is to cultivate an environment where aging is perceived not merely as an unavoidable decline but rather as a manageable process characterized by ongoing growth, health, and fulfillment. Semmelweis University takes a pioneering role by implementing the Semmelweis-EUniWell Workplace Health Promotion Model Program, presenting an opportunity not only to significantly enhance the well-being of its own community but also to catalyze broader transformation in workplace health promotion and healthy aging nationwide. This underscores the centrality of the aging population in the program’s objectives. Within the EUniWell framework, the Semmelweis-EUniWell Workplace Health Promotion Model Program exemplifies the collaborative spirit of the consortium of leading European universities dedicated to the promotion of healthy aging. The Semmelweis-EUniWell Workplace Health Promotion Model Program is fully in harmony with the EUniWell Arena 1 key objective of Community Engagement, specifically contributing to the development of guidelines and work-based learning strategies that promote active aging and support meaningful transitions to retirement among EUniWell employees. By sharing best practices, disseminating knowledge, and fostering innovation across member institutions, the program seeks to develop and implement evidence-based interventions that promote well-being and healthy aging. Additionally, the program collaborates intensively with the WHO Regional Office for Europe and the European Public Health Association’s Healthy Aging Section. These collaborations enhance the program’s capacity to align with international standards and leverage global expertise in promoting healthy aging. By integrating insights and recommendations from these organizations, the Semmelweis-EUniWell Workplace Health Promotion Model Program aims to set a benchmark for workplace health initiatives that can be replicated not only across Hungary but throughout Europe, contributing to the broader goal of enhancing public health and well-being on an international scale. The program’s strategies include detailed cost analysis and support for policy development, ensuring that interventions are both effective and sustainable.

The Semmelweis-EUniWell Workplace Health Promotion Model Program is designed with several key goals in mind, all aimed at fostering a healthier and more supportive environment for employees. This program is informed by the Semmelweis Study, a unique workplace-based longitudinal cohort study designed to elucidate the determinants of unhealthy aging among the employees of Semmelweis University [34]. Firstly, the program seeks to enhance employee well-being by improving the physical, mental, and social health of Semmelweis University staff, with particular attention to optimizing aging trajectories and addressing the needs of aging employees. This involves integrating comprehensive interventions that facilitate healthy aging, including chronic disease prevention and management, ergonomic adjustments, and mental health support. Additionally, the program aims to improve the overall workplace environment, cultivating conditions that promote healthy behaviors and accommodate the needs of an aging workforce, while ensuring freedom from health hazards. By doing so, it fosters a culture of health, encouraging employees to actively engage in health promotion activities and support one another’s well-being. Moreover, the program aspires to contribute to the National Healthy Aging Program by developing a replicable model that can be applied across Hungary. This will support national public health objectives and advance healthy aging goals on a broader scale, ultimately enhancing the well-being of the aging population throughout the country.

To achieve its objectives, the Semmelweis-EUniWell Workplace Health Promotion Model Program is divided into distinct modules, each targeting key factors that impact aging. These modules include physical activity, healthy diet, sleep hygiene, smoking cessation, mental and emotional well-being, work-life balance initiatives, occupational safety, and ergonomics. The physical activity module specifically addresses the sedentary lifestyle of participants. This includes initiatives such as the health promotion program that encourages increased step counts among Semmelweis University employees. This overview focuses on a forthcoming health promotion program, which offers access to micro-gyms, providing a convenient and effective solution for integrating physical activity into the daily routines of university staff. These targeted interventions are designed to promote active lifestyles and contribute to healthier aging.

## Addressing sedentary lifestyles in university and healthcare environments

Sedentary lifestyle has become a pervasive issue in contemporary society, significantly impacting various population groups, including university and healthcare settings. Prolonged periods of physical inactivity are associated with numerous adverse health outcomes, such as cardiovascular diseases, obesity, diabetes, musculoskeletal disorders, and mental health issues like depression and anxiety. These health concerns are particularly pronounced in environments where sedentary behavior is prevalent, such as universities and healthcare institutions.

In university settings, both students and faculty are at high risk for sedentary lifestyles. According to the 2019 European Health Interview Survey, more than 41% of the Hungarian population spends over 7 h per day engaged in sedentary activities, excluding sleeping time. Specific subgroups, such as adolescents (61%), students (58%), and the highly educated workforce (56%), exhibit even higher rates of inactivity [36]. University employees are no exception, with studies indicating significant amounts of occupational sitting time [37]. For instance, research has shown that university employees in administrative roles sit for an average of 501 min per day [38], while those in teaching and research roles also exhibit high levels of sedentary behavior [37, 38].

Similarly, healthcare workers are not immune to the challenges of sedentary lifestyles. A 2018 study from Malaysia found that 45.6% of primary healthcare workers were physically inactive, with 62.8% spending more than four hours per day involved in sedentary activities [39]. Recent studies from the UK also highlight the sedentary nature of healthcare professions, with emergency doctors spending an average of five hours per day sitting [40]. This level of inactivity poses significant risks not only to the physical health of healthcare workers but also to their mental well-being and overall job performance.

The issue of sedentary behavior among university students is equally concerning. A 2020 meta-analysis estimated that college students spend an average of 7.29 h per day engaged in sedentary activities, with computer usage being the largest contributor [41]. Medical students, in particular, exhibit high levels of sedentary behavior, with studies from Spain indicating average sedentary times of 9.63 h per day on weekdays and 6.07 h per day on weekends for female health sciences students [42].

These findings underscore the critical need for targeted health promotion programs aimed at reducing sedentary behavior and increasing physical activity among university and healthcare populations. By addressing the specific challenges faced by these groups, such initiatives can significantly improve their health outcomes and overall quality of life. The Semmelweis-EUniWell Workplace Health Promotion Model Program aims to tackle these issues head on by implementing comprehensive interventions designed to promote active lifestyles and mitigate the adverse effects of sedentary behavior in these settings.

## Micro-gyms: a strategic initiative within the Semmelweis- EUniWell workplace health promotion model program

Workplace physical activity programs have immense potential as a platform for health promotion [43], given the substantial amount of time employees spend at work [44–46]. Recognizing this, the Semmelweis-EUniWell Workplace Health Promotion Model Program includes the innovative implementation of micro-gyms [43] to combat sedentary lifestyles and promote physical activity among university employees.

Micro-gyms, characterized by their compact size and accessibility, offer a promising solution for integrating exercise into the daily routines of employees. These small-scale fitness facilities are strategically placed within workplace environments, such as university departments and healthcare settings [47], making it convenient for staff to engage in regular physical activity without the need for extensive time commitments or travel. The introduction of micro-gyms at Semmelweis University aims to address the specific challenges associated with sedentary behavior among university and healthcare workers. By providing easy access to exercise equipment such as treadmills, stationary bikes, and strength training machines, micro-gyms enable employees to incorporate short, frequent bouts of physical activity into their workday. This approach not only helps reduce sedentary time but also fosters a culture of health and well-being within the workplace [48, 49]. Studies have shown that workplace fitness facilities can significantly increase physical activity levels among employees. For instance, research conducted among university staff revealed a high approval and appeal for workplace fitness rooms as a means to boost physical activity [43]. By aligning with these findings, the Semmelweis-EUniWell initiative seeks to create an environment that encourages spontaneous and regular exercise, thereby promoting healthier lifestyles.

However, research indicates that providing participants solely with a gym membership does not necessarily lead to improvements in fitness levels [50]. This is why we strongly believe that motivational interviewing should play a central role in any health promotion program aimed at increasing physical activity [51]. Motivational interviewing is a collaborative counseling approach that engages employees in active discussions about their health goals and strategies to achieve them [52]. By addressing individual motivations and barriers to exercise, this method has been shown to successfully increase participation in physical activity programs [53]. To enhance the effectiveness of the micro-gym initiative, the program combines micro-gyms with motivational interviewing sessions provided by trained preventive medicine specialists. These sessions are designed to support employees in setting realistic fitness goals, overcoming obstacles, and maintaining their commitment to regular physical activity.

The micro-gym initiative also integrates with other modules of the Semmelweis-EUniWell Workplace Health Promotion Model Program, such as those focusing on healthy diet, mental and emotional well-being, smoking cessation, and ergonomic adjustments. By adopting a holistic approach, the program aims to create a supportive and health-promoting workplace environment that addresses multiple aspects of employee well-being.

The implementation of micro-gyms within the Semmelweis-EUniWell framework represents a strategic effort to reduce sedentary behavior and promote active lifestyles in university and healthcare settings. Through innovative interventions and comprehensive support, the program aspires to improve the health and quality of life of employees, contributing to the broader goals of the National Healthy Aging Program and setting a benchmark for workplace health promotion initiatives within the EUniWell consortium.

## Recommendations to test workplace health promotion programs involving micro-gyms in university settings

A major goal of the Semmelweis-EUniWell Workplace Health Promotion Model Program is to develop evidence-based interventions that are both effective and sustainable. To achieve this, the program incorporates rigorous evaluation methods, including cost–benefit analysis, to ensure that the interventions deliver measurable health benefits while being economically viable. The following method has been developed to test the micro-gym approach in various university settings within the EUniWell framework, with the intention that it can be implemented across all participating institutions in the EUniWell consortium.

The design and implementation of micro-gyms in workplace environments require careful planning to ensure accessibility, efficiency, and employee engagement. The first step in integration is selecting an appropriate location within the workplace, such as repurposing underutilized office space, converting conference rooms, or incorporating modular gym units in shared areas. The design should prioritize flexibility, featuring compact, multi-use equipment such as resistance bands, adjustable weights, and cardio machines that maximize functionality within a small footprint.

The intervention will be designed to include two groups of participants from each university setting. The first group will have access to the micro-gym and receive a single group counseling session focused on the importance of physical activity for healthy aging. The second group will also have access to the micro-gym but will participate in a series of individual motivational interviewing sessions conducted by trained preventive medicine specialists. This dual approach aims to compare the effectiveness of basic gym access with enhanced support through motivational interviewing.

Participants will be recruited from university staff and faculty members. To establish a baseline, data will be collected through online surveys evaluating sociodemographic and health-related variables. Key assessments will include the International Physical Activity Questionnaire (IPAQ) [54] long form to estimate physical activity levels, the 36-item Short Form Health Survey (SF-36) [55] to evaluate both mental and physical well-being, and the Pittsburgh Sleep Quality Index (PSQI) [56] to assess sleep habits.

Both groups will have access to a micro-gym located within their workplace, equipped with various exercise equipment such as treadmills, stationary bikes, and strength training equipment. Participants in both groups will be instructed on the proper use of equipment before the intervention and opportunities provided by the micro-gym. Participants in the first group will then attend a one-time group session that emphasizes the benefits of physical activity and provides general advice on incorporating it into daily routines. In contrast, participants in the second group will engage in five motivational interviewing sessions, approximately 15–30 min each. These sessions will focus on establishing personal fitness goals, developing action plans, overcoming barriers, and maintaining motivation. During the first session, the establishment of a strong doctor-client relationship and the enhancement of motivation are initiated, utilizing various communication tools [57]. Additionally, participants will be also introduced to the task that has to be completed until the next session, which involves journaling their physical activity habits and seeking possible opportunities and occasions that can be used to increase their physical activity. In the second session, the client’s journal will be evaluated. Additionally, goals will also be formulated, specifying the extent to which the client aims to increase their physical activity. It is crucial that the goal is always determined individually, but it is also important to consider that the WHO recommends that individuals aged 18–65 should engage in at least 150 min of moderate-intensity physical activity or a minimum of 75 min of high-intensity physical activity per week [58]. The aim of counselors will be to help patients formulate goals that adhere to these recommendations as closely as possible while also respecting the client’s autonomy. Finally, at the end of the second session, an action plan will be compilated, outlining how the individual plans to achieve the goal they have set. During the third session, client and therapist will examine whether the participant was able to reach their goals. If participants deem it necessary, further adjustments can be made to the plan to further increase the physical activity of participants. However, if individuals are unable to execute the plan or did not manage to increase physical activity to the extent originally planned, the session will focus on normalizing and reframing the experience of failure. Additionally, modifications to the plan may be introduced here too in order to increase the likelihood of success. The fourth session is similar to the third, while the fifth and final session is considered a summary session, during which participants will share what they liked and disliked about the program. They will also reflect on which elements of the plan they can incorporate into their daily lives in the long term.

Post-intervention assessments will be conducted immediately after the intervention, as well as at 1, 3, and 6 months post-intervention to evaluate the sustainability of the program’s impact. The same set of questionnaires (IPAQ, SF-36, PSQI) will be administered to track changes in physical activity, overall well-being, and sleep quality over time.

Data collected from the interventions will be thoroughly analyzed to determine the effectiveness of the micro-gym approach in increasing physical activity and improving health outcomes. The results will be shared among EUniWell member institutions to disseminate best practices and refine the program for broader implementation.

A detailed cost–benefit analysis will be conducted to assess the economic feasibility of the micro-gym interventions. This analysis will consider the initial setup costs of the micro-gyms, operational costs including maintenance and staffing for motivational interviewing sessions, health outcomes such as reductions in healthcare costs due to improved physical activity levels, and the well-being of participants.

By adopting this comprehensive and rigorous methodology, the Semmelweis-EUniWell Workplace Health Promotion Model Program aims to create a robust, evidence-based intervention model that can be replicated across various university settings within the EUniWell consortium and beyond. This collaborative approach will not only enhance the health and well-being of university employees but also contribute valuable insights to the broader field of workplace health promotion.

### Timeline of study

The study on implementing micro-gyms in an occupational setting will unfold over the following timeline:

A **Planning and preparation (months 1–4)**
  **Study design finalization**: finalizing the study protocol, including selection criteria for participants, details of the micro-gym setup, and testing methods, informed consent documents, and other resources necessary for recruitment.
  **Obtaining ethics approval** from designated national organization.
  **Creating marketing materials** (e.g., emails, flyers, meetings, interviews).
B **Phase 2: Recruitment and enrollment (months 4–6)**
  **Staff training**: Ensuring that staff members involved in the study are trained on the micro-gym equipment, study protocols, and assessment methods.
  **Participant recruitment**: Engaging employees through internal communications (e.g., emails, flyers, meetings) to encourage participation in the study.
  **Enrollment of participants**: Enrollment of the first cohort of participants. This period may involve screening for eligibility and obtaining informed consent.
  **Baseline assessments**: Conducting initial assessments for all enrolled participants.
  **Program launch**: Officially launching the micro-gym program, which may include initial orientation sessions for participants on how to use the equipment, schedule their sessions, and track progress.
  **Ongoing participation**: Participants engage with the micro-gym over a defined period, typically 2 moths.
  **End of program**
C **Follow-up phase (6–12 months)**
  **Post-intervention follow-up**: Conducting follow-up assessments to measure the overall impact of the micro-gym program on participants’ health and well-being.
D **Analysis and publication phase (12 months and onwards)**
  **Data collection and analysis**: Aggregating and analyzing data collected throughout the study, including pre- and post-test comparisons for both physical and psychological measures.
  **Publication preparation**: Preparing the manuscript for submission to peer-reviewed journals, including results, conclusions, and future recommendations.

## Long-term considerations

While the initial success of micro-gyms highlights their appeal and effectiveness, long-term adherence and sustainability are crucial for their continued impact. To ensure sustained engagement, micro-gym operators can implement strategies such as employee incentives, structured workplace wellness policies, and personalized membership plans. Providing financial incentives, such as subsidized memberships or performance-based rewards, can encourage consistent participation. Additionally, integrating micro-gym access into workplace wellness programs — offering flexible workout hours, on-site fitness challenges, and team-based activities — can foster a culture of long-term physical activity. Regular engagement through personalized coaching, progress tracking, and community-building initiatives also enhances retention. Employee well-being programs that integrate micro-gyms as part of a company’s health benefits package may also drive long-term usage. Furthermore, leveraging behavioral science insights, such as habit-forming cues and gamification, can improve adherence rates among members. Future research should explore the effectiveness of these strategies in fostering lasting participation. Addressing these factors will help micro-gyms transition from short-term fitness solutions to sustainable wellness hubs.

The long-term effectiveness of micro-gyms can be evaluated through a combination of quantitative and qualitative measures that assess participation rates, health outcomes, employee satisfaction, and workplace productivity. Tracking membership and attendance data over time provides insight into engagement levels and the sustainability of participation. Health metrics such as cardiovascular fitness, body mass index (BMI), and musculoskeletal strength can be measured through periodic assessments, while self-reported surveys can gauge changes in energy levels, stress reduction, and overall well-being. Employee feedback through structured interviews or questionnaires helps assess satisfaction, perceived benefits, and potential areas for improvement. Workplace performance indicators, such as reduced absenteeism, increased productivity, and enhanced job satisfaction, can also provide valuable insights into the broader impact of micro-gyms. Additionally, comparing companies with and without micro-gym access can offer a clearer understanding of their effectiveness in improving employee health and workplace morale. A combination of these evaluation methods ensures a comprehensive assessment of micro-gym success and areas for refinement.

## Benefits and impact of micro-gyms

The positive impact of workplace interventions promoting physical activity on health outcomes of workers is well-documented. Such interventions have been shown to improve fitness, reduce the risk of diabetes, and lead to more favorable lipid profiles and anthropometric measures [59]. Engaging in systematic physical activity is associated with improved quality of life, enhanced insulin sensitivity, additional weight loss, mitigation of adverse effects on bone mass, and improved body composition [60]. Research indicates that exercise performed at the workplace not only enhances physical health but also improves mood, leading to elevated self-perceived work performance [61] and reduced absenteeism [62]. Moreover, regular attendance at workplace fitness centers correlates with reductions in BMI and healthcare costs [63], regardless of the specific activities performed by individuals.

To ensure the efficacy and sustainability of the micro-gym initiative within the Semmelweis-EUniWell Workplace Health Promotion Model Program, comprehensive monitoring approaches will be implemented. One key component of this strategy is the Semmelweis Study, a longitudinal cohort study based at Semmelweis University [34]. This study aims to elucidate the determinants of unhealthy aging and assess the long-term impact of workplace health interventions on employee well-being. The insights gained from the Semmelweis Study will provide a robust evidence base that can inform similar studies at other participating universities within the EUniWell consortium. By leveraging this collaborative framework, the program seeks to develop and refine interventions that can be effectively adapted and implemented across diverse university settings.

A critical aspect of the monitoring approach is the development of a smartphone application tailored for the EUniWell consortium. This app will facilitate closely following lifestyle changes among employees and students at participating institutions. The smartphone application developed for the EUniWell consortium will serve multiple essential functions. First, it will facilitate comprehensive data collection by gathering self-reported information on various health-related behaviors, including physical activity, diet, sleep quality, and mental well-being. This robust data collection will provide a detailed picture of the lifestyle habits of employees and students at participating institutions. In addition to data collection, the app will play a crucial role in motivation. It will offer users personalized feedback and goal-setting tools, along with motivational content designed to encourage sustained engagement in health-promoting activities. This personalized approach aims to keep participants motivated and committed to their health goals over the long term. The app will also be instrumental in monitoring progress. By tracking users’ health behaviors and outcomes over time, it will identify patterns and trends that can inform future interventions and policy decisions. This ongoing monitoring will enable the program to adapt and improve continuously, ensuring its effectiveness and relevance, as well as to identify gaps that may require targeted interventions. By integrating these functionalities, the app will support the comprehensive monitoring and evaluation of all program modules, including those focused on physical activity, diet, mental health, and ergonomics. The strategic use of digital technology will enhance the ability to capture detailed data, engage participants, and provide actionable insights for the continuous improvement of the program. This holistic approach will ensure that the Semmelweis-EUniWell Workplace Health Promotion Model Program remains dynamic and responsive to the needs of its participants, ultimately contributing to better health outcomes and a culture of wellness across the EUniWell consortium.

In summary, the micro-gym initiative within the Semmelweis-EUniWell Workplace Health Promotion Model Program offers substantial benefits for improving employees’ health and well-being. Through rigorous monitoring and the use of innovative digital tools, the program aims to create a scalable, evidence-based model for workplace health promotion that can be implemented across the EUniWell consortium and beyond. This holistic approach not only addresses the immediate health needs of university employees but also contributes to broader public health goals, fostering a culture of wellness and healthy aging throughout Europe.

## Integrating micro-gyms into national health promotion strategies

The scalability of micro-gyms presents a promising avenue for addressing public health challenges, offering a compact size and relatively inexpensive equipment compared to traditional gyms. Their flexibility allows for widespread implementation across diverse workplace settings, both in the state sector and in the private sector. By strategically placing micro-gyms in areas with high population density or limited access to traditional fitness facilities, national health promotion efforts can effectively reach a broad spectrum of the population.

Incorporating micro-gyms into national health promotion programs can significantly help address disparities in physical activity levels and health outcomes among different demographic groups. By providing equitable access to exercise opportunities, micro-gyms ensure that individuals from various socioeconomic backgrounds can benefit from improved physical activity, leading to better overall health outcomes. However, the successful integration of micro-gyms into national health strategies requires careful consideration of several challenges. Funding remains a primary concern, as initial installation costs and ongoing maintenance expenses must be accounted for. Infrastructure development is also crucial, including appropriate space allocation, accessibility, and ensuring facilities meet safety standards. Sustainability is equally critical, as the long-term success of micro-gyms depends on continuous financial investment, user engagement, and effective program monitoring.

To overcome these challenges, collaborative efforts involving government agencies, private sector partners, and community organizations are essential. Government agencies can provide funding and policy support, ensuring that micro-gyms are included in national health promotion plans. Private sector partners can contribute resources and expertise, helping to establish and maintain high-quality facilities. Community organizations can play a vital role in promoting the use of micro-gyms and encouraging local populations to engage in regular physical activity. By leveraging these collaborative efforts, micro-gyms can be effectively integrated into public health initiatives, creating a sustainable model for promoting active lifestyles at scale. The widespread availability of micro-gyms has the potential to reduce physical inactivity, alleviate the burden of chronic diseases, and improve overall population health and well-being. As part of a comprehensive approach to public health, micro-gyms represent a scalable, innovative solution to some of the most pressing challenges faced by modern societies, while contributing to healthier, more resilient communities.

## Challenges, limitations, and recommendations

Implementing micro-gyms and motivational interviewing programs in workplace settings presents several potential obstacles. These challenges include low capacity, the need for trained personnel, time constraints for both employees and employers, and budget constraints for additional staff hours. Addressing these challenges is essential to ensure the success and sustainability of the health promotion initiatives.

### Low capacity and need for trained personnel

One of the primary challenges in implementing micro-gyms and motivational interviewing programs is the limited capacity of micro-gyms, which may not be able to accommodate all interested employees simultaneously. Additionally, there is a critical need for trained personnel to oversee the micro-gyms and conduct motivational interviewing sessions. Addressing these issues requires a multifaceted approach, consisting of the following.

#### Effective scheduling and access management

To ensure that all employees have access to the facilities and support they need, effective scheduling must be implemented. This can involve staggered gym hours, reservation systems, and flexible workout slots that accommodate various work schedules. Digital platforms can be utilized to manage bookings and provide real-time updates on gym availability, ensuring a fair and organized system.

#### Training programs for personnel

Training programs can be developed to certify more personnel in conducting motivational interviews and managing gym facilities. These programs can be tailored to equip staff with the necessary skills to provide support and guidance to employees, enhancing the overall effectiveness of the health promotion initiative. Investing in the professional development of staff ensures that there is a sufficient number of qualified individuals to oversee the program. The challenge of securing trained personnel can be addressed by cross-training existing staff as wellness ambassadors, hiring certified trainers on a part-time basis, or utilizing digital fitness platforms to provide guided workouts. By combining these strategies, micro-gyms can become more accessible and sustainable, ensuring long-term engagement and effectiveness without placing excessive financial or logistical burdens on organizations.

#### Leveraging AI for motivation and support

Artificial intelligence (AI) can play a significant role in addressing the challenges related to low capacity and the need for trained personnel. AI-driven solutions can provide personalized motivation and support to employees, reducing the reliance on human trainers and counselors.

AI-powered chatbots can offer real-time support and motivation to employees, answering questions, providing workout tips, and offering encouragement. These chatbots can be available 24/7, ensuring continuous support. AI could be used in various scenarios, for instance:

- **Personalized recommendations:** AI can analyze individual employee data to offer personalized exercise recommendations and health tips, tailored to their specific needs and goals. This personalized approach can enhance engagement and motivation.
- **Virtual coaching:** AI-driven virtual coaching platforms can simulate the experience of having a personal trainer, guiding employees through workouts, tracking progress, and adjusting plans based on performance and feedback.
- **Gamification:** AI can be used to gamify the fitness experience, creating challenges, setting goals, and offering rewards for achieving milestones. This gamification can make exercise more engaging and fun, boosting motivation.

While AI can significantly enhance motivation and support, it should complement, rather than replace, human interaction. AI can handle routine queries and provide basic guidance, freeing up trained personnel to focus on more complex issues and personalized motivational interviewing sessions. This hybrid approach ensures that employees receive comprehensive support, combining the efficiency of AI with the empathy and expertise of human counselors.

By integrating AI-driven solutions, the program could effectively address the challenges of low capacity and the need for trained personnel. This approach not only ensures that more employees can benefit from the micro-gyms and motivational interviewing sessions but also enhances the overall impact and sustainability of the health promotion initiative.

### Time constraints

Time constraints are another significant barrier, as employees and employers alike may find it difficult to allocate time for physical activity during the workday. Solutions include integrating short, flexible exercise sessions that can be easily incorporated into employees’ schedules. Digital solutions, such as booking systems and reminders, can help manage time effectively and encourage participation. Scheduling conflicts can be alleviated by promoting flexible workout options, such as short, high-intensity sessions, staggered workout times, and integrating fitness into daily routines through walking meetings or active breaks.

### Budget constraints

Budget constraints for additional staff hours required to keep the micro-gym open and clean can pose a significant challenge. However, creative solutions can be implemented to address these financial limitations effectively. One innovative approach is to entrust organizational units, such as academic departments, with the responsibility of running and maintaining the micro-gyms. Due to the small size of these facilities, employees can manage and maintain their own micro-gym, fostering a sense of ownership and responsibility. This not only reduces the need for additional staffing but also presents a valuable team-building opportunity, encouraging collaboration and camaraderie among colleagues. To mitigate budget constraints, micro-gyms can adopt cost-effective solutions such as shared fitness or leveraging existing workplace infrastructure to minimize setup costs. Employers may also seek corporate sponsorships, government wellness grants, or insurance incentives to support fitness programs financially.

### Employee involvement and accessibility

Involving employees in the design process is recommended to ensure the micro-gyms meet their needs and schedules. This participatory approach helps tailor the facilities and programs to the specific requirements of the workforce, increasing the likelihood of sustained engagement. Furthermore, the micro-gyms should be easily accessible from the work area to maximize convenience and usage.

### Health measures and monitoring

Incorporating health measures such as pulse rate monitoring and other options for tracking positive changes resulting from regular training can help sustain employee engagement. Providing feedback on progress and health improvements can motivate employees to continue participating in the program. Alongside physical health measures, motivational interview-based counseling can support employees in setting and achieving their health goals.

### Leadership support and implementation

The strong and unwavering support from Semmelweis University leadership for employee health promotion is pivotal to the program’s successful implementation and adoption. This commitment lays the foundation for prioritizing employee health and well-being across the institution. Securing leadership agreements for employee participation during working hours not only reflects organizational dedication to workplace health promotion but also cultivates a supportive culture that encourages active engagement.

Ongoing consultation and dialogue with leadership throughout the implementation process ensure alignment with institutional goals and the effective allocation of necessary resources. Moreover, active leadership involvement facilitates timely adjustments to the program based on feedback and evolving needs, guaranteeing its long-term effectiveness and sustainability.

In summary, while implementing micro-gyms and motivational interviewing programs presents several challenges, these can be effectively addressed through strategic planning and collaboration. By leveraging digital solutions, involving employees in the design process, and securing support from leadership, the program can create a sustainable and impactful health promotion initiative. The recommended approaches aim to create an environment conducive to healthy behaviors, ultimately improving the well-being of employees and contributing to broader public health objectives.

## Cooperation with EUniWell partner institutions

The Semmelweis-EUniWell Workplace Health Promotion Model Program aims to share its findings and best practices regarding micro-gyms with other institutions across Europe through a combination of research dissemination, collaborative initiatives, and knowledge-sharing platforms. One key strategy is publishing study results in academic journals and policy briefs that highlight the benefits, challenges, and implementation strategies of workplace micro-gyms. Additionally, findings will be presented at EUniWell conferences, workshops, and symposiums to facilitate direct engagement with researchers, policymakers, and institutional leaders.

Collaboration among EUniWell member institutions plays a crucial role in expanding the reach and impact of the program. Joint research projects can explore the effectiveness of micro-gyms in diverse workplace settings, comparing data across different cultural and organizational contexts. Exchange programs and inter-institutional working groups can allow universities and corporate partners to co-develop scalable micro-gym models tailored to specific workplace needs. EUniWell digital platforms can serve as a repository for case studies, training materials, and implementation guidelines, making best practices accessible to institutions across Europe.

Furthermore, EUniWell institutions can collaborate on policy recommendations, advocating for the integration of micro-gyms into European workplace wellness frameworks. By leveraging EU funding opportunities and private-sector partnerships, the program can support pilot projects in different industries, ensuring widespread adoption of workplace micro-gym initiatives. Through these efforts, the Semmelweis-EUniWell program not only advances research on micro-gyms but also fosters a collaborative network dedicated to improving workplace well-being across Europe.

## Conclusion

In conclusion, the Semmelweis-EUniWell Workplace Health Promotion Model Program stands as a pioneering initiative designed to address the pressing challenge of unhealthy aging within Hungary’s workforce. By strategically leveraging the workplace as a platform for health promotion, this program aims not only to enhance the well-being of employees at Semmelweis University and its EUniWell partner institutions but also to catalyze broader transformations in workplace health promotion and healthy aging nationwide. The integration of innovative interventions, such as the combination of micro-gyms and motivational interviewing, is central to the program’s strategy to combat sedentary lifestyles—a major contributor to age-related health issues. By providing convenient access to exercise opportunities and fostering intrinsic motivation through personalized counseling, the program endeavors to empower employees to adopt healthier behaviors and improve their overall health. Moreover, the scalability of micro-gyms presents a promising avenue for national health promotion efforts. These compact and accessible fitness facilities offer equitable access to exercise opportunities across diverse settings, helping to narrow the gap in physical activity levels among different demographic groups. Through concerted efforts and innovative approaches, the Semmelweis-EUniWell Workplace Health Promotion Model Program can advance the goal of healthy aging not only for Semmelweis University employees but for all Hungarian citizens and EUniwell consortium partners. By fostering a healthier, more resilient aging population, this program has the potential to set a benchmark for workplace health promotion initiatives across Europe, contributing to the broader objective of enhancing public health and well-being.
